# Comparison of double-port laparoscopic-assisted extracorporeal suture technique and open surgical repair for Morgagni hernia

**DOI:** 10.55730/1300-0144.5877

**Published:** 2024-05-22

**Authors:** Mehmet SARIKAYA, Fatma ÖZCAN SIKI, Metin GÜNDÜZ, Tamer SEKMENLİ, İlhan ÇİFTCİ

**Affiliations:** Department of Pediatric Surgery, Faculty of Medicine, Selçuk University, Konya, Turkiye

**Keywords:** Congenital hernia, diaphragmatic hernia, laparoscopic, pediatric

## Abstract

**Background/aim:**

We aimed to compare the results of open surgery and double-port laparoscopic-assisted extracorporeal suturing repair for the treatment of Morgagni hernia (MH).

**Materials and methods:**

Twenty-two patients with MH who were operated on in our clinic between January 2012 and January 2023 were included in the study. Patients were divided into two groups according to the surgical technique: open surgery (OS) (n = 14) or laparoscopic surgery (LS) (n = 8). Retrospective comparisons were made between the groups’ demographic information, surgical method used, defect size, operation time, length of hospital stay, costs, postoperative problems, and recurrence.

**Results:**

There were no statistically significant differences between the groups regarding sex, defect size, or costs. The mean age of the patients in the LS group (101 ± 68.3 months) was significantly higher than that of the OS group (23 ± 18.2 months) (p = 0.005). The operation time of the LS group (33.8 ± 3.6 min) was significantly shorter than that of the OS group (50.8 ± 6.5 min) (p < 0.01). Moreover, the LS group’s mean length of hospitalization (1.6 ± 0.9 days) was significantly lower than that of the OS group (2.8 ± 0.7 days) (p = 0.027).

**Conclusion:**

Double-port laparoscopic-assisted extracorporeal suturing repair is a reliable technique preferred over open surgical repair due to its shorter operative time and hospital stay, ease of application, better cosmetic results, and no cost difference.

## 1. Introduction

Morgagni hernia (MH) is a rare congenital anomaly originating from the anteromedial part of the diaphragm and usually occurs on the right side. Abdominal organs are herniated from the defect in the retrosternal area to the thorax. These cases constitute 3%–5% of all diaphragmatic hernias [1,2]. The disease was first described in 1761 by the Italian anatomist Giovanni Battista Morgagni [2]. Since the disease is usually asymptomatic, it is diagnosed late. Frequent lower respiratory tract infections and pain are prominent in symptomatic cases. It is generally diagnosed on radiographs taken during lung infection [3].

The standard approach in cases of MH is open surgical repair. The first successful laparoscopic surgery for MH in a child was documented by Georgacopulo et al. in 1997 [4]. Subsequently, minimally invasive procedures such as laparoscopic-assisted repair were described and successfully applied [5,6]. With the widespread use of laparoscopy after the 1990s, more minimally invasive techniques have begun to be preferred in treating MH [2,7]. We aimed to compare the double-port laparoscopic-assisted extracorporeal suturing repair technique and open surgical repair in MH treatment.

## 2. Materials and methods

After obtaining ethics approval from the relevant local ethics committee (2023/59), patients with MH operated on in our clinic between January 2012 and January 2023 were retrospectively reviewed.

Twenty-two patients operated on for MH were included in this study. The patients were divided into two groups according to the surgical technique: open surgery (OS) (n = 14) or laparoscopic surgery (LS) (n = 8). Retrospective comparisons were made between the groups for demographic information, surgical method used, defect size, operation time, length of hospital stay, costs, postoperative problems, and recurrence. Statistical analysis was conducted using the Mann–Whitney U test for continuous variables and the chi-square or Fisher test for categorical variables.

### 2.1. Laparoscopic surgery technique

**Figure 1:**
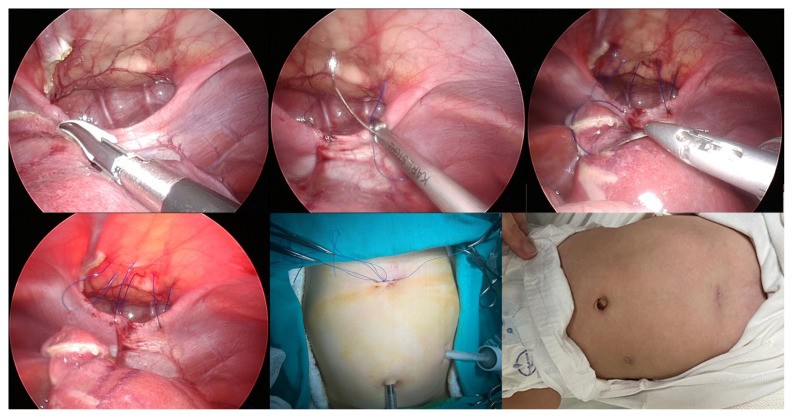
Operation images of laparoscopic-assisted double-port extracorporeal suturing technique: revealing the defect, suturing with 2/0 polypropylene, cosmetic appearance of the abdomen at the end of the operation, and cosmetic appearance at the 2nd week after the operation.

In our preferred approach, the patient is administered general anesthetics and endotracheal intubation is carried out. A 5-mm trocar is inserted into the abdomen with an open technique after making an upper umbilical incision, and pneumoperitoneum is created with 8 mmHg CO_2_ insufflation. Another 5-mm trocar is then placed in the left hypochondrium from the midclavicular line. Intestines encountered during examination in the hernia sac on the anterior thoracic wall are transferred to the abdomen. A 2-mm skin incision is created following the defect’s projection and a 2/0 polypropylene suture is taken through the incision into the abdomen. The needle, caught with a grasper in the abdomen with the tip pointing upwards, is brought out of the abdomen through the same incision, passing through the lower and upper diaphragmatic rims, where the defect is located, respectively. Sequential millimetric incisions are made according to the defect’s width, 3 or 4 sutures are placed in the diaphragm, and the sutures are tied in sequence. Thus, the hernia repair is completed. A 2/0 absorbable suture is used to mend the fascia after the suture knots are implanted under the incision and the trocar is removed. Subcutaneous absorbable 5/0 sutures are used to seal the skin incisions. The steps of the laparoscopic operation are shown in Figures 1a–1d.

### 2.2. Open surgery technique

In our preferred technique, the patient is administered general anesthetics and endotracheal intubation is carried out. The patient is placed in the supine position and the abdomen is entered through a midline incision above the umbilical cord. The defect in the diaphragm is revealed. Herniated organs are taken into the abdomen. In appropriate cases, the hernia sac is excised. The defects are repaired one by one with nonabsorbable 2/0 polypropylene sutures. After surgery, the layers are closed anatomically. Synthetic mesh is not used in any case.

## 3. Results

**Figure 2:**
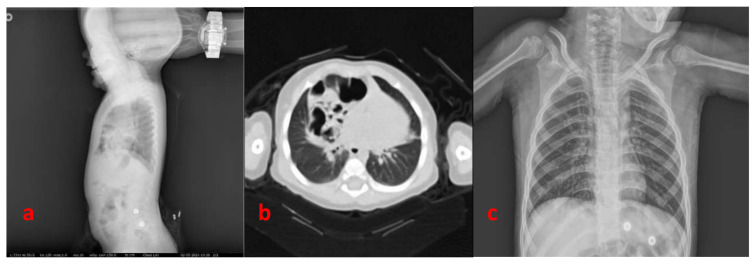
**a)** Lateral chest X-ray of a case of Morgagni hernia; **b)** chest tomography of the same case of Morgagni hernia; c) postoperative chest X-ray with the hernia reduced.

In this study, the data of 22 patients who were operated on for MH were compared statistically. The mean age of the patients was 45.94 ± 52.3 months and the female-to-male ratio was 9:13. Fourteen patients presented with chest infection, which was recurrent in 9 cases. Four patients reported nonspecific respiratory symptoms and asthma-like symptoms. The remaining four patients were diagnosed based on chest X-rays because they had no symptoms. In all of the cases included in this study, noncontrast thorax tomography was performed to confirm the diagnosis and control chest X-rays were obtained in all cases after the operation (Figures 2a–2c). There were 14 patients in the OS group and eight in the LS group. The demographic data, defect size, operation time, costs, postoperative complications, length of hospital stay, and recurrence rates of the patients are detailed in Table 1. There were no statistically significant differences between the groups regarding sex, defect size (p = 0.506), or costs (p = 0.99). The mean age of the patients in the LS group (101 ± 68.3 months) was significantly higher than that of the OS group (23 ± 18.2 months) (p = 0.005). The mean operation time of the LS group (33.8 ± 3.6 min) was significantly shorter than that of the OS group (50.8 ± 6.5 min) (p < 0.001).

Moreover, the LS group’s length of hospitalization (1.6 ± 0.9 days) was significantly lower than that of the OS group (2.8 ± 0.7 days) (p = 0.027). No LS patients needed to transition to open surgery. No intraoperative complications were observed in either group. Adhesive ileus developed in one patient in the OS group in the postoperative 7th month but did not require surgery; it was treated conservatively. Another patient in the same group developed an incisional hernia and was operated on again in the postoperative 10th month. During follow-up, recurrence was detected in one patient in the LS group. This patient underwent open surgical repair 6 months after the initial operation. Upon recurrence, mesh was applied with an open surgical technique in the 9th month after the second operation. There were no problems in the long-term follow-up of the patient. This patient had Down syndrome and immunodeficiency. One patient in the OS group was operated on again 8 months postoperatively due to recurrence of the disease.

Data on the comorbidities of the patients, the sides of the defects, and herniation organs through the defects are given in Table 2. Among the patients included in the study, 10 (45.4%) had trisomy 21, 4 (18.1%) had immunodeficiency, 4 (18.1%) had congenital heart disease, 1 (4.5%) had esophageal atresia-tracheoesophageal fistula, and 2 (9%) had hydrocephalus. The defect was on the right side in 15 (68.2%) cases, on the left side in 4 (18.2%) cases, and bilateral in 3 (13.6%) cases. All patients had colon herniation. Omental hernia was observed in eight patients, cecal hernia in two patients, small intestine hernia in four patients, and liver hernia in two.

## 4. Discussion

MH can be discovered incidentally or as a result of vague gastrointestinal complaints; more commonly, it causes respiratory symptoms, which may be severe during infancy [8]. We observed a similar situation in our series. Most of the patients had a history of recurrent lung infections and, therefore, hospitalization. The disease was detected after an incidental chest X-ray in only four cases.

Another interesting feature of MH is the presence of associated congenital anomalies in 30%–50% of cases [9]. The most common congenital anomalies associated with MH are Down syndrome and congenital heart diseases [9]. In our series, we saw a very high rate of Down syndrome cases (10/22, 45.4%). Congenital heart disease was present in 4 (18.1%) cases, esophageal atresia in 1 (4.5%) case, and hydrocephalus in 2 (9%) cases.

While the mean age of the OS group (23 ± 18.2 months) was consistent with the literature, the mean operative age of the LS group (101 ± 68.3 months) was significantly higher (p = 0.005). In our study, the mean defect sizes of the two groups were similar (OS: 5 ± 1.7 cm, LS: 5 ± 1.5 cm), and these dimensions were consistent with the values of 3–11 cm reported in other studies [7,10].

Although it was reported in the literature that treatment by laparoscopy was less costly than treatment by open surgery [11], no statistically significant cost difference was observed between the groups in our study (OS: 4332 ± 2406 Turkish lira, LS: 4208 ± 1950 Turkish lira; p = 0.99). While performing cost analyses in our research, invoices issued to patients retrospectively were examined and compared using the hospital’s data system. No separate cost calculations were made for laparoscopy materials, which are easily accessible in almost every hospital.

The length of hospital stay was statistically significantly shorter in the LS group (1.6 ± 0.9 days) compared to the OS group (2.8 ± 0.7 days) (p = 0.027), similar to the literature [7,11,12]. Postoperative complications have been reported in the literature at rates of 5%–10% [13]. In our study, while the complication rate was 14.2% in the OS group, no complications were observed in the LS group. There was no statistically significant difference between the two groups regarding postoperative complications (p = 0.27). While the recurrence rate for open surgical repair of MH has been reported to range between 42% to 50% in the literature, this rate has been reported to range between 0% and 42% for laparoscopic repairs [2,14,15]. In our study, recurrence was reported after surgery for one patient in each group and no statistically significant difference was found between the two groups (p = 0.612).

When the operation times of the groups were compared, it was seen that the operation time of the LS group (33.8 ± 3.6 min) was statistically significantly lower than that of the OS group (50.8 ± 6.5 min) (p < 0.001). Based on our findings, it can be said that laparoscopic repair is more advantageous because the operation time and hospital stay were shorter in the LS group and the costs, complications, and recurrence rates did not differ. Better cosmetic appearance is another essential benefit that makes laparoscopic repair advantageous.

Surgery for MH aims to pull the herniated bowel loops into the abdomen and repair the defect. Apart from the classical open surgical procedure, many minimally invasive procedures have been described for defect repair. A few of these minimally invasive methods are patch closure of diaphragm defects, primary suture repair, primary repair with staples, laparoscopic extracorporeal procedures, transabdominal suture placement, knotting devices, hernia sac implantation, robot-assisted laparoscopic repair, and thoracoscopic repair [16–20]. A recent study showed that laparoscopic-assisted MH repair has the advantages of ease of application, shorter operating time, earlier oral feeding time, less pain, shorter hospital stay, and better cosmetic results compared to open surgery [7].

Most laparoscopic interventions described in the literature were performed using three ports with entrance from the umbilicus and both sides [16–20]. In this technique, surgeons generally prefer intracorporeal sutures; the manipulation of surgical instruments is more manageable and the method allows the removal of the hernia sac. The disadvantage of this technique is that the cosmetic appearance is poor since three ports are used. Some surgeons perform MH repair through a single port, but in this technique, one imaging device and two study tools are generally inserted through the same port [21]. Although this technique provides a cosmetic advantage, it is not easy to apply and it is time-consuming due to the overlapping of instruments. Single-port laparoscopic percutaneous MH repair assisted by an optical forceps has been performed in several case series in recent years [11,22]. The advantages of this technique are that it gives perfect cosmetic results and can be performed by a single surgeon without the need for an assistant. The disadvantages of the method are that it is challenging to use simultaneous imaging and surgical instruments, a limited number of surgical instruments can be utilized, the hernia sac cannot be removed, and the defect is more difficult to reveal and that process might be slower. Robotic surgical repair of MH has also been performed in a few case series [23]. The disadvantages of that technique are the high cost of the application, the need to use a large number of ports, the limited number of cases, the long time it takes to set up the devices, and the need for considerable experience.

The double-port technique can be considered an essential innovation in minimally invasive surgery, especially in hernia repairs. This method offers some advantages over traditional open surgery or standard laparoscopic approaches. In more detail, these advantages are as follows:

- Adequate manipulation opportunity: The double-port technique allows the surgeon more mobility in the intrabdominal area. This is a major advantage, especially in difficult anatomical areas or complex hernia repairs.
- Easy determination of defect boundaries: This technique allows the boundaries of the hernia defect to be seen. This allows for more accurate movement during the repair and potentially reduces the risk of recurrence.
- Hernia sac removal: The double-port technique allows for the complete removal of the hernia sac, eliminating the risks of leaving the hernia sac in place, and it can contribute to a healthier healing process.
- Rapid defect repair: Having two ports allows more effective use of the surgical instruments and materials, allowing defect repair to be performed faster.
- However, there are also some disadvantages with this technique:
- Cosmetic disadvantages: An additional incision required for the working port may be a disadvantage, especially for patients concerned about cosmetic results.
- Potential risks of additional incisions: Each additional incision may increase the risk of infection, bleeding, and possible complications during the healing process.

## 5. Conclusion

The double-port laparoscopic-assisted extracorporeal suturing repair technique may offer significant advantages in certain cases. However, it is crucial to weigh the potential advantages and disadvantages of each patient’s unique situation. Surgeons must have sufficient training and experience in this technique, evaluate each case individually, and choose the most appropriate surgical approach.

## Figures and Tables

**Table 1:** Data of patients according to groups.

	Open surgery (OS) group, n = 14	Laparoscopic surgery (LS) group, n = 8	p
F/M	6:8	3:5	
Mean age (months)	23 ± 18.2	101 ± 68.3	0.005
Mean defect size (cm)	5 ± 1.7	5 ± 1.5	0.506
Mean duration of operation (min)	50.8 ± 6.5	33.8 ± 3.6	<0.001
Length of hospital stay (days)	2.8 ± 0.7	1.6 ± 0.9	0.027
Cost in Turkish lira (TL)	4332 ± 2406	4208 ± 1950	0.99
Postoperative complications	2 (14.2%)	0	0.27
Recurrence	1 (7.1%)	1 (12.5%)	0.612

**Table 2:** Clinical features of patients.

Female	9 (40.9%)
Male	13 (59.1%)
Comorbidities	
Down syndrome	10 (45.4%)
Immunodeficiency	4 (18.1%)
Esophageal atresia	1 (4.5%)
Hydrocephalus	2 (9%)
Congenital heart disease	4 (18.1%)
Surgical technique	
Open surgery	14 (63.6%)
Laparoscopic surgery	8 (29.4%)
Side of defect	
Right	15 (68.2%)
Left	4 (18.2%)
Bilateral	3 (13.6%)
Herniated organs	
Colon	22 (100%)
Cecum	2 (9%)
Small intestine	4 (18.1%)
Omentum	8 (36.3%)
Liver	2 (9%)
